# Impact of anaesthetic technique on survival in colon cancer: a review of the literature

**DOI:** 10.1093/gastro/gov001

**Published:** 2015-02-16

**Authors:** F. Jeroen Vogelaar, Daan J. Lips, Frank R.C. van Dorsten, Valery E. Lemmens, Koop Bosscha

**Affiliations:** ^1^Department of Surgery, Jeroen Bosch Hospital, ‘s-Hertogenbosch, the Netherlands; ^2^Department of Surgery, VieCuri Medical Centre, Venlo, the Netherlands; ^3^Department of Anaesthesiology, Jeroen Bosch Hospital, ‘s-Hertogenbosch, the Netherlands; ^4^Department of Research, Netherlands Cancer Registry/Comprehensive Cancer Organisation The Netherlands, Eindhoven, the Netherlands; ^5^Department of Public Health, Erasmus MC University Medical Centre, Rotterdam, the Netherlands

**Keywords:** colon cancer, epidural anaesthesia, immunosuppression, survival

## Abstract

An oncological surgical resection is the mainstay of treatment for potentially curable colon cancer. At the time of surgery, a large fraction of patients do harbour—although not visibly—minimal residual disease at the time of surgery. The immunosuppression that accompanies surgery may have an effect on disease recurrence and survival. Regional or neuraxial anaesthetic techniques like epidural anaesthesia may suppress immune function less than opioid analgesia, by reducing stress response and significantly reducing exposure to opioids. Consistent with this hypothesis, regional anaesthetic techniques have been associated with lower recurrence rates in breast cancer and prostate cancer. Results for colon cancer, however, are contradictory. In this review of the literature we describe all studies addressing the association of the use of epidural anaesthesia and survival in colon cancer surgery.

## Introduction

An oncological surgical resection is the mainstay of treatment for potentially curable colon cancer. However, even in stage I and II colonic cancer, 10–30% will develop recurrence of disease. It is known that, even with the best surgical technique, surgery for cancer is associated with release of tumour cells. Also it is noteworthy that, at the time of surgery, a large fraction of patients do harbour minimal residual disease, although this may not be visible [1].

The idea that surgery itself can promote local cancer recurrence and metastasis is not novel and was described in the 19^th^ century by Velpeau, a French anatomist and surgeon, who noticed that surgical removal of cancer could be associated with the return of the disease and that the operation possibly tended to accelerate tumour growth [2]. Whether this results in recurrence of clinical cancer or metastasis depends largely on the balance between the tumour’s ability to spread and the immunosurveillance of the patient [3]. General anaesthesia and surgical stress may suppress immunity by directly affecting the immune system or activating the hypothalamic-pituitary-adrenal axis and the sympathetic nervous system [4]. Pre-operative and post-operative opioids may inhibit cellular and humoral immune function in humans, and morphine itself might have a pro-angiogenic effect that promotes tumour growth [5].

Regional or neuraxial anaesthetic techniques may suppress immune function less than opioid analgesia by reducing stress response and significantly reducing exposure to opioids. Consistent with this hypothesis, regional anaesthetic techniques have been associated with lower recurrence rates of breast- and prostate cancer [6, 7]. Results for colon cancer, however, are contradictory [8]. In this review of the literature we describe all studies addressing the association of the use of epidural anaesthesia (EA) and survival in colon cancer surgery.

## Methods

Relevant studies were sought in the Pubmed database (starting date January 1990 up to June 2014) using search terms as follows: (i) “regional anesthesia” or “regional anaesthesia” or “regional analgesia” or “anesthetic technique” or “anaesthetic technique”, (ii) “recurrence” or “survival” and (iii) “colorectal cancer” or “colon cancer”. Also, we searched “related citations” and reference lists to identify other articles. Only full papers published in the English language were included. We did not define a minimum of patients to qualify for inclusion in the analysis.

The following information was gathered from the articles: (i) number of included patients, (ii) design of the study, (iii) age, (iv) type of tumour (colon and/or rectal), (v) tumour stage, (vi) follow-up, and (vii) effect of anaesthetic technique on overall survival and cancer recurrence.

## Results

A total of seven studies was found addressing the impact of EA on survival in colorectal cancer surgery [8–14]. Table 1 shows the characteristics of each of these.

**Table 1.** Characteristics of described studies

First author	Year of publication	Study design	No. of patients	Mean age (years)	Cancer type	Stage	Follow up (years)	OS benefit from EA	RFS benefit from EA
Christopherson	2008	Prospective	177 EA: 85 No EA:92	69	Colon	I–IV	Up to 10 years	Better OS in stage I–II; No benefit in stage III–IV	Not assessed
Gottschalk	2010	Retrospective	509 EA: 256 No EA: 253	64	Colon (*n = *283) Rectal (*n = *202) ‘others’ (*n = *25)	I–IV	Median, 1.8	Not assessed	Better RFS in older patients (≥65 years old)
Gupta	2011	Retrospective	655 EA: 562 No EA: 93	73 (colon) 69 (rectal)	Colon (*n = *360) Rectal (*n = *295)	I–III	Mean, 2.6	Better OS in rectal cancer	Not assessed
Myles	2011	Prospective	112^a^ EA: 58 No EA: 54	71 (epidural) 70 (no-epidural)	Colon	I–III	Up to 12 years	No benefit	No benefit
Day	2012	Retrospective	424 EA: 107 (251 including spinal) No EA: 173	72 (epidural) 70 (PCA) 70 (spinal)	Colon (*n = *314) Rectal (*n = *110)	I–III (?) Not clearly described	Median, 3.1 (epidural) 2.3 (PCA) 1.4 (spinal)	No benefit	No benefit
Cummings	2012	Retrospective	42,151 EA: 9670 No EA: 32,481	≥66	Colon (*n = *33 390) Rectal (*n = *8761)	I–III	Up to 14 years	Better OS	No benefit
Holler	2013	Retrospective	749 EA: 442 No EA: 307	Not available	Colon (*n = *369) Rectal (*n = *380)	I–IV	Up to 8 years	Better OS (especially in ASA classification 3 to 4)	Not assessed

^a^As a part of 446 patients undergoing major abdominal surgery for different types of cancer

EA = epidural anesthesia; OS = overall survival; PCA = patient-controlled analgesia; RFS = recurrence-free survival

### Prospective studies

Two of these seven studies were prospective. Christopherson *et al*. studied long-term survival after resection of colon cancer as a sub-analysis of a prospective randomized study. This Veterans Affairs Co-operative Study No. 345 was initially designed to compare the short-term effect of general anaesthesia with and without epidural anaesthesia and analgesia supplementation in patients undergoing abdominal surgery. Randomization was stratified for type of surgery, age and cardiac risk [13]. The second prospective study was the Multicentre Australian Study of Epidural Anaesthesia and Analgesia in Major Surgery (the MASTER trial), primarily designed to compare adverse outcomes in high-risk patients managed for major surgery with epidural block or alternative analgesic regimens with general anaesthesia in a multicentre randomized trial [14].

#### Overall survival

Christopherson *et al*. found a better overall survival in the first 1.5 post-operative years in 177 colon cancer patients for stage I–II patients with a mean age of 69 years old who received EA [HR 0.21 (95% CI 1.40–15.42); *P = *0.012]. Nevertheless, the type of anaesthesia did not appear to affect long-term survival [13]. Of 446 patients in the MASTER trial, with a mean age of 70–71 years undergoing major abdominal surgery for different types of cancer, 112 underwent surgery because of stage I–III colon cancer. They did not find that the use of EA (*n = *58) was associated with improved overall survival [14].

#### Disease-free survival

Of the two prospective studies, only the MASTER trial made a disease-free survival analysis. EA in this study was not associated with improved disease-free survival.

### Retrospective studies

Five retrospective studies were included in this review. Of all reviewed literature, the largest retrospective study was the Surveillance, Epidemiology, and End Results (SEER)-based study, with a large cohort of 42 151 patients aged 66 years or older and diagnosed with non-metastatic colorectal carcinoma [8]. Holler *et al.* studied 749 stage I–IV colorectal cancer patients in their large retrospective analyses [12]. The Swedish study of Gupta *et al.,* of a total of 655 colorectal patients with a mean age of 69 (rectal cancer) and 73 (colon cancer) years old, excluded emergency operations, laparoscopically-assisted resections and stage IV in their analysis [11]. Day *et al*. studied colon and rectal cancer patients with a mean age of 70 (no epidural) and 72 (epidural) years old [9]. All underwent a laparoscopic resection in this study. Patients received either an epidural (*n = *107), spinal block (*n = *144), or morphine, patient-controlled analgesia (PCA) (*n = *173) for their primary post-operative analgesia. Gottschalk *et al.* analysed stage I–IV patients (*n = *509), of which there were 283 with colon cancer, 202 with rectal cancer and 25 ‘others’ [10].

#### Overall survival

Four of the retrospective studies assessed overall survival analysis. The large SEER-based study found a significant association between EA and improved overall survival (HR 0.91 (95% CI 0.87–0.94); *P < *0.001) [8]. A significantly better overall survival was also found by Holler *et* *al*. in 442 patients who received EA (5-year survival rate with EA was 62%, but only 54% without EA; HR 0.73; *P < *0.02) [12]. The positive impact in this study was the most significant in high-risk patients defined as American Association of Anaesthesiologists (ASA) classification 3–4 (*P = *0.006) [12]. The Swedish study found a reduction in all-cause mortality in rectal cancer patients (*n = *295) who received EA (HR 0.45 (95% CI 0.22–0.90); *P = *0.025) [11]. Day *et al*. found no overall survival difference in their analysis [9].

#### Disease-free survival

In the study by Gottschalk *et al*. during median follow-up of 1.8 years, EA was associated with a lower cancer recurrence in 248 patients older than 64 years (*P = *0.01), but not in younger patients (*n* = 261) [10]. The SEER-based study adjusted for demographic and clinical covariates and did not find a significant difference in the odds of recurrence between the groups during a mean follow-up of 5 years [8]. Also no recurrence-free survival difference was found in the study by Day *et al.* [9].

## Discussion

Because the anticancer immune response is a primary determinant of cancer progression, it is logical to hypothesize that interventions aimed at reducing exposure to immunosuppressive factors would improve patient outcomes after a potentially curative cancer resection. Although EA is theoretically supposed to be a favourable immune-modulating intervention, not all studies show a consistent beneficial effect from EA in colon cancer patients. Seven studies are included in this review, of which two had a prospective design. Four of the seven studies showed an overall survival benefit in patients receiving EA although, in three of these, the effect was only seen in subgroups (stage I–II in the first one-and-a-half year post-operative, rectal cancer patients and ASA 3–4 patients). A cancer recurrence survival benefit from EA was found in one study—in older patients. One of the studies found no negative effect of EA on recurrence-free or overall survival.

Because of the retrospective nature of five of the seven studies, unrecorded factors may have influenced survival: for example, potentially important treatment characteristics like the use of chemotherapy and radiation are missing in all studies except Gupta *et al.* [11]. Although, in some studies, tumour grade is known [10, 12, 14], other tumour-specific characteristics that influence prognosis—such as lymphangio-invasion, tumour perforation and microsatellite instability—are unknown.

It is hypothesised that volatile anaesthesia and opioids may have a negative effect on the anti-cancer immune system, especially ‘natural killer’ (NK) cells [5, 15, 16]. EA might reduce the requirement of volatile anaesthesia, and obviate the need for opioid administration. None of the studies give detailed information about the analgesic and anaesthetic techniques currently in use.

In two of the seven studies, only colon cancer patients were studied [13, 14], while four studies analysed colorectal cancer patients as one group [8–10, 12]. Only Gupta *et* *al.* made a sub-analysis for colon and rectal cancer [11]. As a possible explanation for the better survival for rectal cancer patients with EA in their study, they suggest that rectal cancer may be more susceptible to the protective effect of regional analgesia than colonic cancer. No specific pathophysiological mechanism for this hypothesis is given.

NK cells are chiefly responsible for cytotoxic activity against spontaneously derived tumour cells. Data from the literature have shown that both the total and the relative numbers of circulating NK cells are greater in healthy elderly people than in young adult ages. The age-related increase of NK-cell numbers can be regarded as a compensatory mechanism for the decreased cytolytic activity per cell in elderly subjects. Total NK-cell cytotoxicity is steady, but, the NK-cell cytotoxicity on a ‘per cell’ basis is impaired [17]. Gottschalk *et* *al.* suggested that the benefit of EA to the immune system (and especially the NK cells) might be greater in older subjects, because they only found a recurrence-free survival benefit from EA in patients older than 64 years [10]. Although specific changes of the effect of EA on NK cells in elderly subjects might play a role in different results of the studies, the possible underlying mechanism needs to be further clarified in future studies.

Different surgical techniques may also have influenced the results, especially the laparoscopic *vs.* open approaches. The study by Day *et al.* looked only at patients receiving laparoscopic colorectal resections [9]. The reason why no survival advantage was identified with the use of regional analgesia in this study may be due to the laparoscopic approach. Laparoscopy is known to reduce the degree of immunosuppression that occurs during the post-operative period, when compared with that of an open colorectal resection [18]. If a significant preservation of immune function occurs with laparoscopic colorectal resection, the choice of analgesia used may be less important. On the other hand, a large number of trials comparing laparoscopic and open surgery for colorectal cancer can be identified in the literature. A recent meta-analysis stated that laparoscopic surgery for colon cancer does not differ from open surgery in terms of overall survival [19]. None of the prospective studies in our review stratified for the type of surgery (laparoscopic *vs.* open).

Finally, the effect of EA might not only be anti-tumour, but also favour other mechanisms. Although cancer recurrence will determine survival to a large extent, other putative mechanisms include a reduction in perioperative cardiac-, respiratory- and thromboembolic events, but this effect mainly influences short-term survival [20]. A recent Cochrane review concluded that, compared with general anaesthesia, a central neuraxial block may reduce the 0–30-day mortality for patients undergoing surgery with intermediate-to-high cardiac risk [21].

In conclusion, this review of seven heterogeneous studies shows that the association between EA and survival of colon and rectal cancer is not clear, as conflicting results are described in the literature—although none of the studies showed a negative influence of EA on survival. Randomized, prospective, well-stratified studies are needed to determine whether the association between EA and (cancer-specific) survival is causative.

*Conflict of interest statement:* none declared.
